# Incidence of Spinal CSF Leakage on CT Myelography in Patients with Nontraumatic Intracranial Subdural Hematoma

**DOI:** 10.3390/diagnostics11122278

**Published:** 2021-12-06

**Authors:** Hyo Jin Kim, Joon Woo Lee, Eugene Lee, Yusuhn Kang, Joong Mo Ahn

**Affiliations:** 1 Department of Radiology, Seoul National University Bundang Hospital, 82 Gumi-ro 173 Beon-gil, Bundang-gu, Seongnam 13620, Korea; khjsm4@gmail.com (H.J.K.); eugene801027@gmail.com (E.L.); yskang0114@gmail.com (Y.K.); joongmoahn@gmail.com (J.M.A.); 2 Department of Radiology, Seoul Metropolitan Government-Seoul National University Boramae Medical Center, Seoul National University College of Medicine, 20 Boramae-ro 5-gil, Dongjak-gu, Seoul 07061, Korea; 3 Department of Radiology, Seoul National University College of Medicine, Seoul 03080, Korea

**Keywords:** intracranial subdural hematoma, spontaneous intracranial hypotension, CT myelography, epidural blood patch

## Abstract

The aim of the present study was to demonstrate the incidence of spinal cerebrospinal fluid (CSF) leaks in patients with nontraumatic intracranial subdural hematoma (SDH) and determine clinical parameters favoring such leaks. This retrospective study was approved by the institutional review board. Patients diagnosed with nontraumatic intracranial SDH who underwent computed tomography (CT) myelography between January 2012 and March 2018 were selected. 60 patients (male: female, 39:21; age range, 20–82 years) were enrolled and divided into CSF leak-positive and CSF leak-negative groups according to CT myelography data. Clinical findings were statistically compared between the two groups. Spinal CSF leak was observed in 80% (48/60) of patients, and it was significantly associated with an age of <69 years (*p* = 0.006). However, patients aged ≥69 years also had a tendency to exhibit spontaneous intracranial hypotension (SIH)-induced nontraumatic intracranial SDH (60.87%; 14/23). Therefore, CT myelography is recommended to be performed for the evaluation of possible SIH in patients with nontraumatic intracranial SDH, particularly those aged <69 years. Patients aged ≥69 years are also good candidates for CT myelography because SIH tends to occur even in this age group.

## 1. Introduction

Nontraumatic intracranial subdural hematoma (SDH) can be induced by a variety of causes. Hypertensive cortical artery rupture [1], middle meningeal artery aneurysm rupture [2], idiopathic bleeding, coagulopathy, oncological bleeding, and cocaine-induced bleeding have been reported as causative factors for acute SDH [3]. Causative factors for chronic SDH include stretching of bridging veins due to extensive brain atrophy, fragile neovasculatures associated with neomembrane formation after subdural hygroma or acute SDH [4]. For both acute and chronic SDH, conservative management (reversal of anticoagulation and prophylactic anticonvulsants) or surgical treatment (hematoma evacuation) is used depending on the patient’s symptoms and extent of hematoma [4].

Spontaneous intracranial hypotension (SIH) can also result in nontraumatic SDH. SIH is a disorder characterized by decreased cerebrospinal fluid (CSF) volume and pressure, and it is caused by a persistent CSF leak through a dural defect along the neuraxis [5]. Spontaneous focal dural thinning and dehiscence are common causes of CSF leaks. Degenerative abnormalities of the spine, including disk protrusions and osteophytes, may also result in thecal sac tears. Although some authors also report CSF–venous fistula as one of the causes, this remains a topic of speculation [6]. A CSF leak can result in downward traction on the brain, causing headaches, subdural fluid collection, and possible brain herniation [5]. Occasionally, tearing of the bridging veins results in SDH [7]. In such cases, hematoma evacuation prior to repair of the CSF leak may be ineffective, and untreated downward traction can lead to further postoperative accumulation of SDH [8]. Therefore, for optimal treatment of some patients with SDH, clinicians should recognize the possibility of SIH as a cause of hematoma and search for CSF leaks requiring repair with procedures such as an epidural blood patch (EBP) [8]. When dealing with patients with SDH, the differential diagnosis of SIH should be emphasized, particularly in younger patients, patients without a history of head trauma, and patients with postural headaches [9]. Currently, computed tomography (CT) myelography is considered the gold standard for the initial evaluation of SIH [10,11,12] because it offers superior anatomic details [13].

Beck [14] conducted a prospective study and reported that spinal CSF leaks were present in 25.9% of nongeriatric patients (≤60 years) with chronic SDH. However, to the best of our knowledge, there is no other structured study on the incidence of imaging-confirmed CSF leaks in patients with SDH. Since 2012, neurosurgeons at our institution request CT myelography to rule out SIH in patients with nontraumatic SDH without any explainable cause. If a CSF leak is detected on CT myelography, EBP is performed. Accordingly, we have observed a higher rate of CSF leaks than that reported in Beck’s study. Therefore, we designed the present study to demonstrate the incidence of spinal CSF leaks and determine potential clinical parameters favoring such leaks in patients with nontraumatic SDH.

## 2. Materials and Methods

### 2.1. Patients

This retrospective study was approved by the institutional review board of our hospital, which waived the need for informed consent because of the retrospective study design. Neurosurgeons at our institution requested CT myelography to rule out SIH in patients with nontraumatic SDH without any explainable cause. Two research assistants went through the hospital’s electronic medical records between January 2012 and March 2018 then retrieved the details of patients diagnosed with SDH and subjected to CT myelography. The following inclusion criteria were applied to the patients identified from the medical records: no history of trauma, absence of coagulopathy according to a coagulation panel and platelet count measurements, absence of intracranial mass lesions susceptible to spontaneous bleeding, no history of drug abuse, performance of follow-up brain imaging at three months after treatment for SDH, and age >18 years. Eventually, 60 patients (male:female, 39:21; mean age, 58.65 ± 15.52 years; range, 20–82 years) were included. According to the previously written reports of CT myelography, the patients were divided into CSF leak-positive and CSF leak-negative groups.

### 2.2. Retrospective Review of Electronic Medical Records and Imaging Findings

A radiologist who was blinded from CT myelography reports retrospectively reviewed the patients’ demographic data, Glasgow coma scale scores, anticoagulant use, presence or absence of orthostatic headache at the first visit, laterality of SDH and the degree of midline shift in initial images, treatment the patients underwent, and follow-up findings in brain images taken within, and at three months [15,16] after treatment for SDH. The radiologist also recorded whether recurrence had developed on follow-up brain images. We defined ‘recurrence’ of SDH as a subsequent increase in hematoma volume in subdural space and compression of the brain surface after treatment by referring to the previous several studies [16,17].

### 2.3. Statistical Analysis

To determine variables that were significantly associated with CSF leaks, the medical records for CSF leak-positive and leak-negative groups were analyzed using chi-square tests/Fisher’s exact tests for discrete variables and *t*-tests for continuous variables. A *p*-value of <0.05 was considered statistically significant.

### 2.4. Our Routine CT Myelography Procedure & Interpretation

In a fluoroscopy room, the patient is placed on a radiolucent table in the lateral decubitus position, with the right side up and knee flexed. Using a midline interlaminar approach between the third and fourth lumbar vertebrae under fluoroscopy guidance, a trained musculoskeletal radiologist inserts a 22-gauge spinal needle into the CSF space. Following the confirmation of CSF drainage via the spinal needle, 15 cc of contrast medium (OMNIPAQUE 300, Amersham Health, Princeton, NJ, USA) is slowly injected through the needle. When the contrast medium reached the spinal canal at the atlantooccipital level, the patients are transferred to the CT unit for whole-spine imaging, and the acquired data are presented in axial, sagittal, and coronal planes (Brilliance 64 CT scanner, Philips Healthcare, Best, Netherlands; helical; beam collimation, 64 × 0.625 mm; kVp, 120; mAs, 250; pitch, 0.798; rotation time, 0.5 s; thickness, 2 mm; increment, 1 mm).

Trained musculoskeletal radiologists immediately interpret the obtained images to confirm the presence of contrast media leakage (=CSF leak) exists. A positive CSF leak is defined as extrathecal CSF accumulation at any level. Meningeal diverticula are not considered as CSF leaks because single or multiple nerve root sleeve diverticula of various sizes and configurations can be seen as incidental findings [6].

### 2.5. Our Routine EBP Procedure

If CSF leaks are confirmed by CT myelography, targeted EBP with autologous blood is performed under fluoroscopic guidance. If there are multiple leaks, EBP is performed at the mid-level of the leaks in order to ensure the widest coverage. Generally, 10 cc of blood is used for each targeted level, and a total of up to 20 cc of blood is used.

## 3. Results

In total, 48 of the 60 (80%) patients exhibited CSF leaks on CT myelography. Differences in parameters between the leak-positive and leak-negative groups are shown in Table 1.

The proportions of patients aged <69 years (*p* = 0.006) were significantly higher in the leak-positive group than in the leak-negative group. However, patients aged ≥69 years also had a tendency to exhibit SIH-induced SDH (14/23; 60.87%).

**Figure 1:**
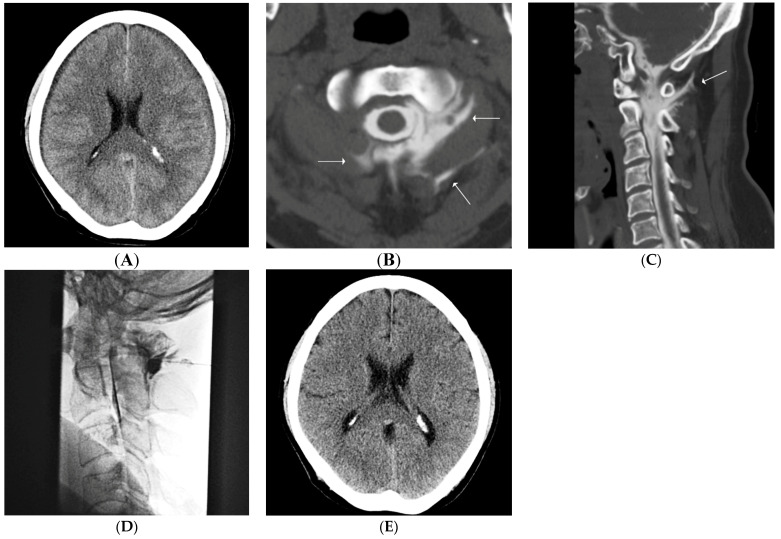
(**A**–**E**) Findings for a representative case involving a 53-year-old man with nontraumatic intracranial subdural hematoma (SDH). The patient presented with a chief complaint of headache not related to a specific posture. (**A**). Brain computed tomography (CT) image shows bilateral intracranial SDH. He was referred to us for CT myelography and epidural blood patch (EBP) the day after undergoing burr-hole trephination and hematoma removal. (**B**,**C**). Axial and sagittal CT myelography images show a large amount of cerebrospinal fluid leaks at the level of the C1/2 left extradural space (solid arrows). (**D**) EBP is performed at the C1/2 level. (**E**). Follow-up brain CT performed after 3 months shows no evidence of SDH.

**Figure 2:**
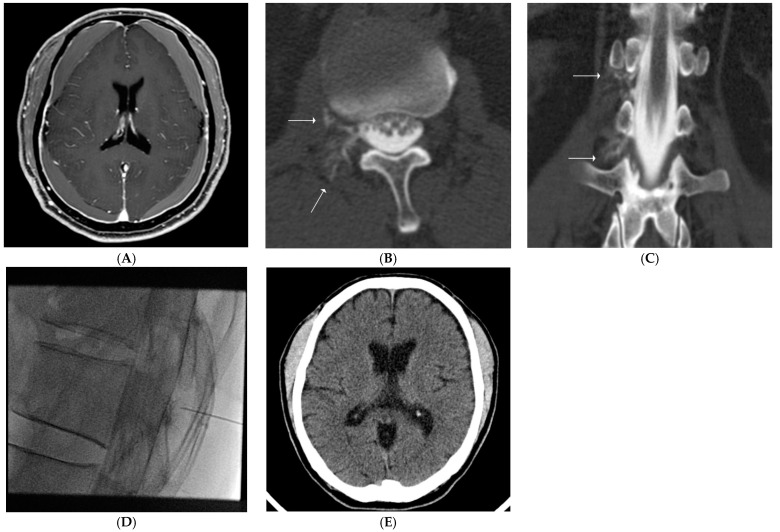
(**A**–**E**). Findings for a representative case involving a 70-year-old man with nontraumatic intracranial subdural hematoma (SDH). The patient presented with a chief complaint of headache not related to a specific posture. (**A**). Contrast-enhanced, fat-suppressed T1 weighted image shows bilateral nontraumatic SDH with pachymeningeal thickening and enhancement. (**B**,**C**). Axial and coronal computed tomography (CT) myelography images show cerebrospinal fluid leaks at the level of the T12/L1 and the L1/2 right extradural space (solid arrows). (**D**). An epidural blood patch is performed at the T12/L1 level. Intrathecal staining is present due to a previously injected contrast agent for CT myelography. (**E**). Follow-up brain CT performed after 3 months shows no evidence of SDH.

Targeted EBP was performed for all 48 leak-positive patients, with 31 undergoing surgical removal of hematoma as well as EBP. From these 31 patients, three developed recurrence repeatedly after several surgeries but showed complete resolution following one or two EBP procedures; 10 patients developed recurrence after a single EBP procedure and necessitated repeated EBP from one to three times with surgical evacuation (eight patients needed one surgery and two patients required two surgeries) until there was no recurrence; 18 underwent surgery and EBP at about the same time (within 24 h), and among them, one had additional surgery due to recurrence; meanwhile, the other 17 did not have to undergo further invasive procedures (Figure 1 and Figure 2A–E).

**Figure 3:**
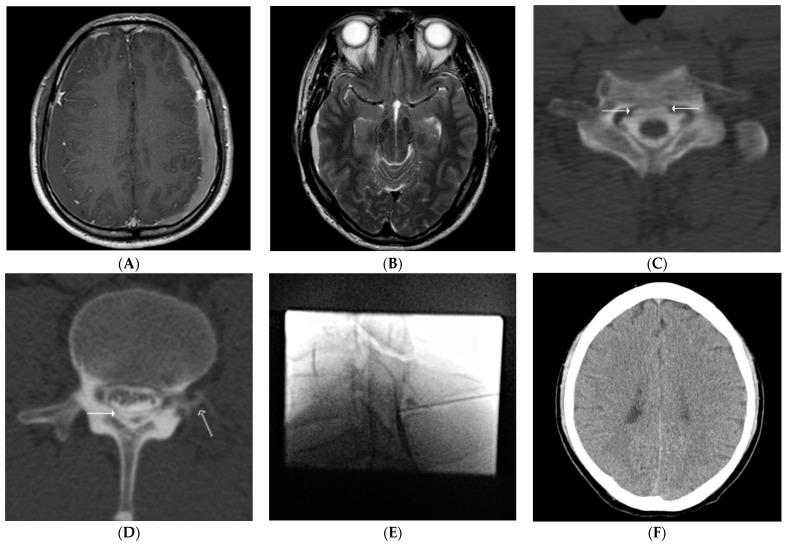
(**A**–**F**). Findings for a representative case involving a 41-year-old man with nontraumatic intracranial subdural hematoma (SDH). The patient presented with a chief complaint of headache not related to a specific posture. (**A**). Contrast-enhanced, fat-suppressed T1 weighted image shows bilateral nontraumatic SDH with pachymeningeal thickening and enhancement. (**B**). T2 weighted image at the initial presentation shows cisternal obliteration. (**C**,**D**). Computed tomography (CT) myelography performed to evaluate possible spontaneous intracranial hypotension shows cerebrospinal fluid leaks at the level of the C6/7 ventral epidural space (solid arrows, (**C**)), the L2/3 dorsal epidural space (a solid arrow, (**D**)), and the left extraforaminal space (a hollow arrow, (**D**)). (**E**). Subsequent epidural blood patch is performed at the C6/7 and L2/3 (not shown) levels. (**F**). Follow-up brain CT performed after 3 months shows no evidence of SDH.

Of the 17 patients who underwent EBP only, 14 developed no recurrence (Figure 3A–F) and three developed recurrence after a single EBP procedure; the latter three patients underwent a second EBP procedure and did not develop recurrence thereafter.

Of the 12 leak-negative patients, eight underwent surgical removal of hematoma, one underwent both surgical removal of hematoma and EBP, and one underwent EBP alone. The latter two patients underwent empirical nontargeted EBP at the discretion of neurosurgeons, although they showed negative CT myelography findings. The remaining two patients only received conservative management. All 12 leak-negative patients showed hematoma resolution with no recurrence after treatment.

A total of 40 patients required surgery for nontraumatic SDH. In the leak-positive group, a total of 31 patients underwent surgery; burr-hole trephination and hematoma removal in 25 patients, and craniotomy in 6 patients. In the leak-negative group, a total of 9 patients underwent surgery; burr-hole trephination and hematoma removal in 7 patients and craniotomy in 2 patients. The reoperation rate after EBP was 7.5%.

## 4. Discussion

The present study revealed that the incidence of spinal CSF leaks was 80% in patients with nontraumatic SDH. An age of <69 years was significantly associated with the presence of CSF leaks, although patients aged ≥69 years also tended to exhibit SIH-induced SDH (60.87%). A total of 40 patients required surgery. The reoperation rate after EBP was 7.5%.

The standard management strategy for SDH generally involves decompression surgery or conservative care with close observation depending on the age of the hematoma, degree of the midline shift, clot thickness, and neurological status [4,18]. However, a different treatment strategy is necessary for the sealing of CSF leaks in patients with SIH-induced SDH [19,20,21,22]. Therefore, for optimal treatment in some patients with SDH, recognition of the possibility of SIH as a cause of SDH is important [8]. In a study of the incidence of imaging-confirmed CSF leaks in patients with SDH, Beck [14] found that spinal CSF leaks were present in 25.9% nongeriatric patients (≤60 years) with chronic SDH. In the present study, we detected leaks in 80% patients with nontraumatic SDH who underwent CT myelography to rule out suspected SIH. This rate is considerably higher than that reported by Beck. Additionally, SIH-induced SDH was also seen in older age groups than in the study by Beck. These suggest that clinicians need to broaden their scope of doubt regarding SIH and expand the indications for studies to detect CSF leaks, such as CT myelography, in patients with nontraumatic SDH.

It would be interesting to know why the leak-positive and leak-negative groups showed no significant difference with regard to the presence of orthostatic headache, one of the most famous clinical findings of SIH. One reason could be that orthostatic headache may be seen even in the presence of relatively small leaks that are not large enough to be detected on CT myelography. In such cases, magnetic resonance myelography with intrathecal gadolinium, which is considered an effective medium for the detection of low flow leaks, can be considered [6]. However, considering that all of our leak-negative patients who did not receive EBP showed no recurrence or aggravation of symptoms, we recommend that slow, intermittent, or small leaks that are not detectable on CT myelography may not always require identification or EBP.

The percentage of reoperation in patients with SDH varies depending on the literature. According to one study, the reoperation rate for chronic SDH is 9.4 to 19.5% [15]. In the current study, 40 patients underwent surgical removal of hematoma, and among them, three patients needed reoperation (7.5%) after EBP. This figure is lower than previously reported, and it suggests that timely EBP may allow a good outcome in patients with SIH-induced SDH.

This study has several limitations. First, although the leak-positive rate revealed by imaging studies was higher than that in the previous study, there could have been differences in the CT myelography protocol between the previous study and our study in terms of the radiation dose, slice thickness, pitch, and reconstruction interval. Because there was no exact match between the test conditions, the comparison between the two studies may have limited value. Second, this was a retrospective study, and the sample size was relatively small. Larger-scale, prospective studies may be needed to further clarify our findings.

## 5. Conclusions

In conclusion, the findings of the present study suggest that CT myelography is recommended to be performed for the evaluation of possible SIH in patients with nontraumatic SDH, particularly those aged <69 years. Patients aged ≥69 years are also good candidates for CT myelography because SIH tends to occur even in this age group.

## Figures and Tables

**Table 1:** Statistical Analysis of Parameters for Patients with Cerebrospinal Fluid Leaks and Those without Leaks among a Cohort of Patients with Nontraumatic Intracranial Subdural Hematoma.

	Leak (+) Total N = 48	Leak (−) Total N = 12	*p*-Value
Male sex, n (%)	31 (64.58)	8 (66.67)	1.000
Mean age ± standard deviation	56.85 ± 15.50	65.83 ± 13.93	0.165
Age < 69, n (%)	34 (70.83)	3 (25.00)	0.006
Wafarin, n (%)	0 (0.00)	1 (8.33)	0.200
Aspirin, n (%)	8 (16.67)	1 (8.33)	0.671
Clopidogrel, n (%)	3 (6.25)	0 (0.00)	1.000
Orthostatic headache, n (%)	12 (25.00)	1 (8.33)	0.628
Glasgow coma scale score of 15, n (%)	48 (100.00)	12 (100.00)	N/A
Unilateral subdural hematoma, n (%)	9 (18.75)	5 (41.67)	0.13
The degree of midline shift (mm ± standard deviation)	3.34 ± 3.47	3.82 ± 4.46	0.29

## Data Availability

The data presented in this study are available on request from the corresponding author. The data are not publicly available due to privacy.
